# Reliability of the Dynamic Foot Index for Observational Assessment of Foot Motion During Gait

**DOI:** 10.1002/pri.70196

**Published:** 2026-03-20

**Authors:** Guilherme Augusto Santos Araujo, George Schayer Sabino, Francisco D'Paula Vitor Ferreira, Matheus Hissa Lourenço Ferreira, Renan Alves Resende

**Affiliations:** ^1^ Faculdade de Sete Lagoas (FACSETE) Sete Lagoas Minas Gerais Brazil; ^2^ Department of Physical Therapy Graduate Program in Rehabilitation Sciences School of Physical Education Physical Therapy and Occupational Therapy Universidade Federal de Minas Gerais (UFMG) Belo Horizonte Minas Gerais Brazil; ^3^ Faculdade Ciências Médicas de Minas Gerais (FCMMG) Belo Horizonte Minas Gerais Brazil; ^4^ Department of Sport Sciences School of Physical Education Physical Therapy and Occupational Therapy Universidade Federal de Minas Gerais (UFMG) Belo Horizonte Minas Gerais Brazil

**Keywords:** foot, gait, physical therapy modalities, reproducibility of results

## Abstract

**Background and Purpose:**

Reliable clinical tools for observational assessment of dynamic foot motion during gait are limited. Although three‐dimensional motion analysis is considered the reference standard, its cost and complexity restrict routine clinical use. The Dynamic Foot Index (DFI) is a simple, low‐cost observational tool designed to assess foot motion during gait.

**Methods:**

This methodological study investigated the intra‐ and inter‐rater reliability of the Dynamic Foot Index (DFI) in healthy adults. Forty‐nine participants (98 feet) walked barefoot on a treadmill at a self‐selected speed while being recorded from a posterior view using a smartphone camera. Two experienced physical therapists independently analyzed video recordings of one complete stride per limb, classifying foot motion across three stance‐phase subphases (loading response, midstance, and push‐off) using a three‐point ordinal scale. Intra‐rater reliability was assessed after a 30‐day interval. Weighted Kappa coefficients with 95% confidence intervals and absolute percentage agreement were calculated.

**Results:**

Intra‐rater reliability ranged from moderate to substantial across gait subphases (weighted Kappa = 0.54–0.80), with higher agreement observed during loading response and push‐off. Inter‐rater reliability ranged from fair to substantial (weighted Kappa = 0.28–0.68), with lower values during midstance. Absolute agreement ranged from 65% to 92%, depending on the subphase and limb assessed.

**Discussion:**

The Dynamic Foot Index (DFI) demonstrated moderate to substantial intra‐rater reliability and variable inter‐rater reliability across stance‐phase subphases in healthy adults. These findings support the use of the Dynamic Foot Index (DFI) as a clinically feasible observational tool for longitudinal assessment of foot motion during gait, particularly when applied by the same evaluator.

## 1 Introduction

During the stance phase of gait, foot pronation and supination movements play a fundamental role in the interaction with the ground across the different subphases of the gait cycle (Kumar et al. 2018; Mousavi et al. 2024). During loading response, pronation allows the foot to become more flexible, facilitating the absorption of ground reaction forces and adaptation to different surfaces (Michaud 1997). This movement is mainly characterized by calcaneal eversion, medial rotation, and plantarflexion of the talus, associated with lowering of the medial longitudinal arch (Ito et al. 2017). In contrast, during midstance and push‐off, the foot must acquire greater rigidity to act as an efficient lever in transmitting propulsive force to the ground, thereby contributing to forward progression of the body. In these subphases, supination predominates and is characterized by calcaneal inversion, lateral rotation, and dorsiflexion of the talus, in addition to elevation of the medial longitudinal arch (Lundgren et al. 2008; Blackwood et al. 2005).

Alterations in the magnitude, duration, or timing of these movements during the stance phase of gait have been associated with the development and recurrence of different musculoskeletal conditions, such as medial tibial stress syndrome and patellofemoral pain (Neal et al. 2014; Newman et al. 2013; Barton et al. 2015). In this context, the clinical identification of atypical foot movement patterns may assist physical therapists in decision‐making regarding the need to investigate associated musculoskeletal factors, such as excessive forefoot varus (Monaghan et al. 2014) or weakness of the intrinsic foot muscles (Headlee et al. 2008; McKeon et al. 2015). Based on this assessment, targeted interventions become possible to modify the identified movement pattern and manage the mechanical demands imposed on the musculoskeletal system (Mousavi et al. 2024).

The assessment of foot movement during gait can be performed in a valid and reliable manner using three‐dimensional motion analysis systems (Daly et al. 2009). However, the high cost of these systems, combined with the need for specialized infrastructure and advanced technical training, limits their routine use in clinical settings. In contrast, technological advances in smartphone cameras, together with the use of free video analysis software such as Kinovea, have enabled two‐dimensional motion assessments with acceptable validity and reliability for different functional tasks (Fernández‐González et al. 2020; Puig‐Diví et al. 2019). Previous studies have shown that qualitative video‐based analyses can provide clinically relevant information about the movement behavior of lower limb segments, such as the trunk, hip, and knee, during functional tasks such as squatting and single‐leg jumping (Kingston et al. 2020).

Despite this evidence, there remains an important gap regarding the specific observational clinical assessment of foot movements during the stance phase of gait in adults. In the absence of dedicated dynamic instruments, static measures of foot posture, such as the Foot Posture Index (Redmond et al. 2006; Aquino et al. 2018; Behling and Nigg 2020) or the measurement of medial longitudinal arch height (Williams and McClay 2000; Nilsson et al. 2012), are commonly used as indirect strategies to infer dynamic foot behavior. However, evidence indicates that the correlation between static posture and dynamic foot pattern during gait is, at best, weak to moderate (Behling and Nigg 2020; Hamill et al. 1989), which limits the clinical usefulness of these approaches when used in isolation.

In light of this gap, the development of simple, low‐cost, observation‐based clinical instruments capable of assessing dynamic foot behavior during gait becomes particularly relevant for routine clinical practice. The Dynamic Foot Index (DFI) was designed as a structured observational tool to support clinical gait assessment, with a primary intended application in longitudinal follow‐up conducted by the same clinician rather than diagnostic classification. By standardizing visual observation across key subphases of stance, the DFI aims to inform clinical reasoning, assist in identifying atypical movement patterns that may warrant further assessment, and support monitoring of changes over time or in response to intervention. Accordingly, the aim of this study was to investigate the intra‐ and inter‐rater reliability of the DFI during gait.

## 2 Methods

### 2.1 Study Design and Ethical Aspects

This methodological study was designed to evaluate the reliability of the Dynamic Foot Index (DFI) as a structured observational tool for clinical gait assessment, reflecting its intended use in routine practice rather than diagnostic classification. Intra‐ and inter‐rater reliability were assessed in accordance with the Consensus‐based Standards for the Selection of Health Measurement Instruments (COSMIN) guidelines (Mokkink et al. 2018), with reporting guided by the COSMIN Risk of Bias checklist for reliability (Checklist S1), as well as the principles of the Declaration of Helsinki and established recommendations for observational studies. The study protocol was approved by the local Institutional Research Ethics Committee (00890818.8.000.5149).

### 2.2 Participants

The sample consisted exclusively of healthy young adults, reflecting the exploratory nature of this initial reliability study and the intention to first evaluate the performance of the DFI in a population with low variability in gait patterns.

Sample size was determined based on recommendations from the Consensus‐based Standards for the Selection of Health Measurement Instruments (COSMIN), which consider a sample of 50–99 participants adequate for reliability studies of measurement instruments (Mokkink et al. 2018). Based on this criterion, 50 participants were initially recruited. However, one participant (2%) was lost due to a logistical reason unrelated to the experimental protocol, resulting in a final sample of 49 participants (98% retention). Although the final sample size was slightly below the COSMIN recommendations, minimal sample loss and high data completeness are unlikely to have affected the reliability estimates.

The final sample included 49 participants (28 women and 21 men), totaling 98 feet assessed. Participants had a mean age of 23.72 ± 4.2 years, mean height of 1.68 ± 0.11 m, mean body mass of 63.92 ± 11.28 kg, and a mean body mass index (BMI) of 22.37 ± 3.07 kg/m^2^.

Inclusion criteria were: (1) age between 18 and 50 years; (2) absence of neurological or rheumatological diseases; (3) BMI < 30 kg/m^2^; (4) no current use of biomechanical orthoses; (5) no history of surgical interventions in the lower limbs; and (6) absence of musculoskeletal injuries in the six months prior to data collection. Volunteers reporting pain or discomfort in the lower limbs that could interfere with the experimental procedures were excluded. The sample was recruited for convenience.

### 2.3 Instrument: Dynamic Foot Index (DFI)

The DFI is an observational scale inspired by the Foot Posture Index, developed to assess foot kinematics in the frontal plane during gait. The instrument evaluates the relative movement between the leg, calcaneus, and forefoot across three gait subphases, defined according to (Perry and Burnfield 2024): (Kumar et al. 2018) Loading Response (Mousavi et al. 2024), Midstance, and (Michaud 1997) Push‐off (Mokkink et al. 2018).

Scoring uses a three‐point Likert scale to classify foot behavior:

−1 (Supination): Calcaneal inversion or foot adduction.

0 (Neutral): Small magnitude of movement or neutral alignment.

+1 (Pronation): Calcaneal eversion or foot abduction.

### 2.4 Data Collection Procedures

Participants walked barefoot on a treadmill. To preserve the ecological validity of gait, a self‐selected speed was used, determined according to the protocol described by Holt (1999). In this procedure, participants progressively adjusted the treadmill speed until reporting comfort, with the corresponding speed considered after three consecutive consistent trials.

Image acquisition was performed using a smartphone camera (Apple iPhone XS Max), configured at a resolution of 3840 × 2160 pixels (4K) and a sampling frequency of 60 frames per second (fps). The device was positioned posterior to the participant, approximately 1 m from the treadmill and at the height of the knee joint line, in order to ensure adequate framing of the leg and foot segments. After gait stabilization, 30 continuous seconds of video were recorded.

### 2.5 Video Processing and Analysis

Videos were processed using the free software Kinovea (version 0.9.5), which is widely used for two‐dimensional kinematic analyses. For each participant, isolated video clips representing one complete stride of each limb (right and left) were created to standardize the analyzed material. The analysis of a single complete stride per limb was adopted to standardize observational conditions across participants and evaluators and to reflect typical clinical gait assessment, which is commonly based on brief visual observation rather than averaging across multiple gait cycles. This approach has been adopted in previous observational gait reliability studies and allows consistent comparison of scoring decisions between raters.

Analyses were performed by two independent raters, both physical therapists with more than 14 years of clinical experience in foot biomechanics. Prior to analysis, the raters participated in a structured familiarization and calibration session lasting approximately 60 min. During this session, 10 representative video clips were jointly reviewed in slow motion to align the interpretation of the DFI categories across gait subphases. Training focused on standardizing visual attention to predefined anatomical landmarks (e.g., calcaneal orientation and relative alignment of the rearfoot), as well as clarifying the operational definitions of each DFI category as described in the instrument manual. No quantitative thresholds were applied; instead, representative examples were used as visual anchors to support consistent categorical judgment. Following this calibration session, all video analyses were performed independently and in a blinded manner, with no further interaction between the raters.

### 2.6 Reliability Procedures

The reliability of the DFI was assessed through intra‐ and inter‐rater analyses. For intra‐rater reliability, one rater reassessed the same video clips after a 30‐day interval, without access to the initial analysis results, in order to minimize memory effects and ensure independence between assessments.

Inter‐rater reliability was determined by comparing the independent assessments performed by the two raters. Analyses were conducted separately for each lower limb (right and left) and for each of the three subphases of the stance phase of gait assessed by the DFI (loading response, midstance, and push‐off).

Each video clip was classified according to the DFI criteria using a three‐point ordinal scale (−1, 0, and +1). All assessments were performed using the same video clips, ensuring that observed differences reflected exclusively intra‐ or inter‐rater variability.

## 3 Statistical Analysis

Descriptive analysis was used to characterize the sample based on anthropometric data, with continuous variables presented as mean and standard deviation.

Intra‐ and inter‐rater reliability of the DFI, for each subphase of the stance phase of gait, was assessed using the weighted Kappa coefficient, which is appropriate for ordinal data, with 95% confidence intervals. Kappa values were interpreted according to the criteria proposed by Viera and Garrett (2005): slight (0.01–0.20), fair (0.21–0.40), moderate (0.41–0.60), substantial (0.61–0.80), and almost perfect (0.81–1.00) (Viera and Garrett 2005).

Additionally, absolute percentage agreement was calculated to complement the interpretation of reliability coefficients, particularly in the context of low variability in movement patterns.

All statistical analyses were performed using R software (version 4.4.1) (R Core Team 2025), within the RStudio environment (Posit team 2024).

## 4 Results/Findings

Forty‐nine healthy young adults participated in the study, including 28 women and 21 men, totaling 98 feet assessed. Participants had a mean age of 23.72 ± 4.2 years, mean height of 1.68 ± 0.11 m, and mean body mass of 64.02 ± 11.03 kg. All volunteers completed the experimental protocol without any adverse events.

### 4.1 Intra‐Rater Reliability

Intra‐rater reliability of the DFI ranged from moderate to substantial, with weighted Kappa values between 0.54 and 0.80, depending on the gait subphase and the side assessed. Four of the six analyses showed reliability classified as substantial. The highest agreement values were observed in the push‐off subphase of the right foot (Kappa = 0.80) and during loading response for both feet (Kappa = 0.77 for the right foot and 0.71 for the left foot).

Absolute intra‐rater percentage agreement ranged from 78% to 92%. The analyses with the lowest intra‐rater reliability were observed during midstance of the right foot (Kappa = 0.56; agreement = 78%) and during push‐off of the left foot (Kappa = 0.54; agreement = 82%).

Detailed intra‐rater reliability results are presented in Table 1.

**TABLE 1:** Intra‐rater reliability of the Dynamic Foot Index.

Gait subphase	Foot	Weighted Kappa	Absolute agreement (%)
Loading response	Right	0.77	84
Midstance	Right	0.56	78
Push‐off	Right	0.80	92
Loading response	Left	0.71	84
Midstance	Left	0.71	86
Push‐off	Left	0.54	82

### 4.2 Inter‐Rater Reliability

Inter‐rater reliability ranged from fair to substantial across gait subphases, with weighted Kappa values between 0.28 and 0.68. Three of the six analyses showed reliability classified as moderate. The highest reliability values were observed during the push‐off subphase of the right foot (Kappa = 0.68) and during the loading response of the left foot (Kappa = 0.61).

The midstance subphase showed the lowest levels of inter‐rater reliability, particularly for the left foot (Kappa = 0.28; agreement = 69%). Absolute inter‐rater percentage agreement ranged from 65% to 80%.

Complete inter‐rater reliability results are presented in Table 2.

**TABLE 2:** Inter‐rater reliability of the Dynamic Foot Index.

Gait subphase	Foot	Weighted Kappa	Absolute agreement (%)
Loading response	Right	0.56	67
Midstance	Right	0.47	78
Push‐off	Right	0.68	80
Loading response	Left	0.61	67
Midstance	Left	0.28	69
Push‐off	Left	0.49	65

### 4.3 Distribution of DFI Scores

The distribution of DFI scores revealed a high prevalence of neutral classifications (score 0) across all gait subphases. Pronation (+1) and supination (−1) scores occurred less frequently, particularly during the midstance subphase, reflecting the homogeneity of the movement pattern in the assessed sample.

**FIGURE 1:**
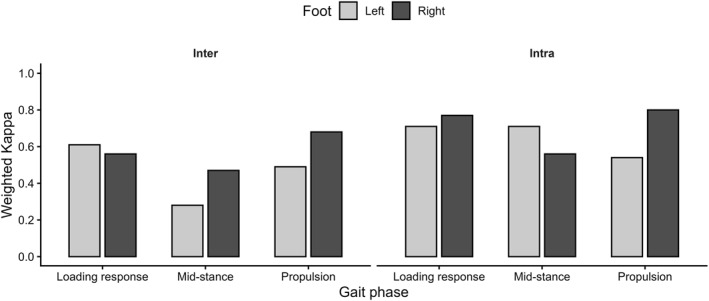
Intra‐ and inter‐rater reliability of the Dynamic Foot Index across gait subphases.

Intra‐ and inter‐rater reliability values of the DFI, expressed by weighted Kappa coefficients for each subphase of the stance phase of gait (loading response, midstance, and push‐off) and for the right and left limbs separately, are presented in Figure 1.

## 5 Discussion

The present study investigated the intra‐ and inter‐rater reliability of the DFI during the stance phase of gait in healthy young adults. The results demonstrated that the DFI shows predominantly moderate to substantial intra‐rater reliability, supporting its clinical applicability for longitudinal monitoring of foot behavior when used by the same evaluator. In contrast, inter‐rater reliability showed greater variability across the analyzed subphases, with lower performance observed during midstance. These findings align with the original purpose of the DFI as a structured observational instrument intended to support clinical reasoning and follow‐up assessments, rather than diagnostic classification.

Intra‐rater reliability values, expressed by weighted Kappa coefficients ranging from 0.54 to 0.80, are consistent with previous findings from observational scales applied to gait analysis. Mackey et al. (2003), when evaluating the Observational Gait Scale, reported similar levels of intra‐rater agreement, reinforcing that visually based observational instruments can demonstrate adequate temporal stability when applied in a standardized manner (Mackey et al. 2003). From a clinical perspective, this consistency is particularly relevant, as it allows score variations over time to be interpreted as reflecting real changes in motor behavior rather than fluctuations due to measurement error. Accordingly, the DFI appears useful as a follow‐up tool, assisting clinicians in decision‐making regarding the need to investigate musculoskeletal parameters potentially associated with changes in foot behavior, such as intrinsic muscle function (Headlee et al. 2008; McKeon et al. 2015).

Regarding inter‐rater reliability, weighted Kappa values ranged from fair to substantial, with better performance in the loading response and push‐off subphases compared with midstance. This pattern should be interpreted in light of the characteristics of the sample, which consisted exclusively of young and asymptomatic individuals. Sim and Wright (2005) described the so‐called Kappa paradox, in which a high prevalence of a dominant category can artificially reduce coefficient values even when absolute agreement is relatively high (Sim and Wright 2005). In the present study, the predominance of neutral classifications resulted in an imbalanced marginal distribution, which may have contributed to underestimation of Kappa values in some subphases. As a methodological limitation, alternative agreement coefficients less sensitive to prevalence, such as Gwet's AC1, were not calculated and could complement the interpretation of inter‐rater reliability in the context of imbalanced marginal distributions (Wongpakaran et al. 2013). From a clinical perspective, these findings suggest that caution is warranted when DFI scores obtained during midstance are compared across different evaluators. In settings involving multiple clinicians, additional training, calibration, or consensus procedures may be necessary to ensure consistent interpretation, particularly in populations with subtle or near‐neutral foot alignment.

Despite the known sensitivity of Kappa coefficients to category prevalence, weighted Kappa was selected as the primary reliability statistic because it is the most widely used and recommended measure for ordinal observational scales in clinical research (Sim and Wright 2005; Landis and Koch 1977). In the present study, weighted Kappa was interpreted alongside absolute agreement to provide complementary information on chance‐corrected agreement and raw concordance between evaluators. The inclusion of absolute agreement values was intended to aid interpretation in subphases with imbalanced category distributions, in which Kappa coefficients may underestimate agreement. Nevertheless, the absence of prevalence‐adjusted coefficients such as Gwet's AC1 represents a methodological limitation and should be addressed in future investigations, particularly in more heterogeneous or symptomatic populations (Gwet 2008).

The lower reliability values observed during midstance should be interpreted in light of the biomechanical characteristics of this subphase and the observational design of the instrument (Mahoney et al. 2019; Leardini et al. 2007). During midstance, pronatory foot behavior results predominantly from the interaction between deformation of the medial longitudinal arch and internal tibial rotation, components that manifest mainly in the sagittal and transverse planes and show less visual expression in a posterior view (Mahoney et al. 2019); Leardini et al. 2007). In young and asymptomatic individuals, these manifestations tend to be even more subtle, with alignment remaining close to neutral, which reduces visual discriminability between adjacent categories (Leardini et al. 2007). Moreover, the DFI was conceived as a two‐dimensional posterior‐view observational tool, aligned with clinical approaches previously described in the literature, prioritizing clinical feasibility and low cost, which implies reduced sensitivity to alterations that are not clearly expressed in the frontal plane (Dao et al. 2023; Matsuzaka et al. 2025). Thus, lower reliability values during midstance reflect biomechanical and observational limitations inherent to the assessed subphase, rather than inconsistency of the instrument or evaluators (Matsuzaka et al. 2025; Brown et al. 2008). This pattern reinforces that DFI reliability is dependent on the gait subphase, being higher when mechanical signs are more evident in the frontal plane, such as during loading response and push‐off (Brown et al. 2008).

In the DFI protocol, assessment was performed exclusively from a posterior view. Evidence indicates that the visual expression of pronatory behavior varies according to plantar arch morphology. Cornwall and McPoil (2011) demonstrated that in feet with a lower medial longitudinal arch, pronation tends to manifest more clearly through calcaneal eversion, whereas Resende et al. (2015) observed that in feet with a higher arch, arch mobility and tibial rotation may represent more relevant indicators of this behavior (Cornwall and McPoil 2011; Resende et al. 2015). In addition, Behling and Nigg (2020) emphasized that the relationship between static arch posture and foot dynamics during gait is variable, which complicates inference of three‐dimensional foot behavior from a single plane of observation (Behling and Nigg 2020). Therefore, the absence of a simultaneous lateral view in the present protocol may have limited the ability to discriminate subtle movement alterations during midstance, contributing to the lower inter‐rater agreement observed in this subphase (Maykut et al. 2015; Pipkin et al. 2016; Brunnekreef et al. 2005).

Additionally, although the evaluators had extensive clinical experience in gait analysis, assessment using segmented two‐dimensional videos imposes perceptual demands that differ from continuous clinical observation (Brunnekreef et al. 2005; Eastlack et al. 1991). In this regard, Viehweger et al. (2010) highlighted that clinical experience alone does not guarantee high reliability in video‐based observational analysis when available visual parameters are restricted. This finding reinforces the importance of specific and standardized training for inter‐rater application of the DFI, particularly in subphases in which the relevant movement occurs predominantly outside the frontal plane (Viehweger et al. 2010). This reinforces that inter‐rater application of the DFI should be accompanied using structured familiarization and calibration procedures, especially when the instrument is used for comparative purposes across evaluators.

Overall, the results indicate that the DFI demonstrates methodological robustness for intra‐rater application, constituting a consistent and low‐cost clinical tool for longitudinal monitoring of foot behavior in populations with low variability in gait patterns. Inter‐rater reliability was shown to be dependent on the analyzed subphase, with lower agreement during midstance, reflecting both sample homogeneity and biomechanical limitations inherent to two‐dimensional posterior‐view analysis. These findings do not limit the clinical utility of the instrument but clearly delineate its scope of application and provide objective support for future refinements, such as incorporating additional views that may increase the observational sensitivity of the DFI.

The exclusive inclusion of healthy young adults represents an important limitation of this study. Although appropriate for an initial reliability assessment, the homogeneity of the sample likely contributed to the high prevalence of neutral classifications and may have influenced inter‐rater reliability estimates, particularly during midstance. Therefore, the present findings should not be generalized to symptomatic, older, or more heterogeneous clinical populations without further investigation.

The decision to analyze a single stride per limb represents a methodological choice aligned with the observational nature of the DFI and its intended clinical use. Although averaging scores across multiple strides may reduce within‐subject variability and potentially influence reliability estimates, this approach would also diverge from typical clinical gait assessment and increase analysis time. Future studies may explore whether averaging across multiple gait cycles affects the reliability or interpretability of DFI classifications, particularly in more heterogeneous or symptomatic populations.

From a measurement development perspective, the present study represents an initial step in establishing the measurement properties of the DFI by demonstrating its reliability under standardized observational conditions. To support broader clinical adoption, future studies should evaluate additional properties in accordance with COSMIN recommendations, including validity testing against three‐dimensional motion analysis systems, responsiveness to clinically meaningful changes following intervention, and estimation of measurement error parameters such as the standard error of measurement and minimal detectable change. In more advanced stages of instrument development, the investigation of minimal clinically important differences may also be warranted, particularly in symptomatic populations. Together, these steps will help position the DFI within a coherent and progressive measurement framework for the observational assessment of dynamic foot behavior during gait.

The DFI demonstrated moderate to substantial intra‐rater reliability in healthy young adults, supporting its use as a simple, low‐cost clinical tool applicable to the longitudinal monitoring of foot behavior during gait when used by the same evaluator. Inter‐rater reliability showed greater variability across gait subphases, with lower agreement observed during midstance, a subphase sensitive to limitations inherent to two‐dimensional posterior‐view analysis. Thus, the DFI appears suitable for intra‐rater clinical applications, whereas comparisons between evaluators should be interpreted with caution. Future studies should investigate the application of the instrument in populations with greater variability in gait patterns and explore the incorporation of additional views to enhance the observational sensitivity of the DFI.

### 5.1 Implications of Physiotherapy Practice

The findings of this study have direct implications for physiotherapy practice, particularly in settings where access to three‐dimensional motion analysis is limited. The Dynamic Foot Index (DFI) provides clinicians with a simple, low‐cost, and time‐efficient observational tool to assess foot motion during gait using standard video recordings. The demonstrated moderate to substantial intra‐rater reliability supports the use of the DFI for longitudinal monitoring of foot behavior when applied by the same evaluator, allowing changes over time to be interpreted with greater confidence. In clinical practice, the DFI may assist physiotherapists in identifying atypical movement patterns during specific stance‐phase subphases, informing clinical reasoning, guiding further biomechanical assessment, and supporting decision‐making regarding interventions targeting foot and lower limb function. Given the variability observed in inter‐rater reliability, particularly during midstance, comparisons between different evaluators should be interpreted with caution unless standardized training and calibration procedures are implemented. Overall, the DFI represents a feasible adjunct to routine gait assessment in physiotherapy, especially for follow‐up evaluations and outcome monitoring in contexts that prioritize practicality and accessibility.

## Funding

The study was supported by Coordenação de Aperfeiçoamento de Pessoal de Nível Superior.

## Ethics Statement

This study was approved by the Institutional Research Ethics Committee of the Universidade Federal de Minas Gerais (UFMG) (CAAE: 00890818.8.000.5149), approved on December 1, 2021.

## Consent

All participants provided written informed consent prior to participation in the study.

## Conflicts of Interest

The authors declare no conflicts of interest.

## Supporting information


Supporting Information S1

## Data Availability

The datasets generated and analyzed during the current study are available from the corresponding author upon reasonable request.
