# Supplementary material for: Reliability of the Dynamic Foot Index for Observational Assessment of Foot Motion During Gait
Source: Physiother Res Int. 2026 Mar 20;31(2):e70196. doi: 10.1002/pri.70196 (PMC13003379; doi:10.1002/pri.70196)
Supplement: Supplementary file 1 — Supporting Information S1 [file PRI-31-e70196-s001.docx]

**Checklist S1. COSMIN Risk of Bias checklist for reliability**

**PART A – Comprehensive research question**

| **Item** | **Response** |
| --- | --- |
| Name of the outcome measurement instrument | Dynamic Foot Index (DFI) |
| Version / operationalization of the measurement instrument | Observational assessment of foot motion during gait using posterior-view video recordings captured with a smartphone camera during treadmill walking at self-selected speed. |
| Construct measured | Dynamic foot motion during gait. |
| Measurement property of interest | Reliability (intra-rater and inter-rater). |
| Component(s) of the instrument that were repeated | Assignment of the score based on the same video recordings. |
| Source(s) of variation | Raters (inter-rater reliability) and time (intra-rater reliability). |
| Patient population | Healthy adults. |

**PART B – Risk of Bias assessment (Reliability)**

| **COSMIN Standard** | **Rating** | **Justification** |
| --- | --- | --- |
| Stability of participants | Very good | Healthy adults with no intervention between measurements; same video recordings used. |
| Appropriate time interval | Very good | Thirty-day interval to minimize recall bias while ensuring participant stability. |
| Similar measurement conditions | Very good | Identical video recordings, equipment, and scoring protocol used for all assessments. |
| Administration of measurements | Not applicable | Only score assignment was repeated using recorded videos. |
| Assignment of the score | Very good | Scores were assigned independently and blinded to previous ratings. |
| Other important methodological flaws | Very good | No other important methodological flaws identified. |
| Appropriate statistical methods | Very good | Weighted Kappa coefficients and percentage agreement were used for ordinal outcomes. |
| Sample size | Adequate | Sample size consistent with COSMIN recommendations for reliability studies. |

**Measurement Error**

| **Item** | **Rating** | **Justification** |
| --- | --- | --- |
| Measurement error | Not applicable | Measurement error was not evaluated in this study. |
